# High-Density-Nanotips-Composed 3D Hierarchical Au/CuS Hybrids for Sensitive, Signal-Reproducible, and Substrate-Recyclable SERS Detection

**DOI:** 10.3390/nano12142359

**Published:** 2022-07-10

**Authors:** Hao Fu, Weiwei Liu, Junqing Li, Wenguang Wu, Qian Zhao, Haoming Bao, Le Zhou, Shuyi Zhu, Jinglin Kong, Hongwen Zhang, Weiping Cai

**Affiliations:** 1 Key Laboratory of Materials Physics, Institute of Solid State Physics, Hefei Institues of Physical Science (HFIPS), Chinese Academy of Sciences, Hefei 230031, China; haofu@issp.ac.cn (H.F.); baohm@issp.ac.cn (H.B.); zhoule@issp.ac.cn (L.Z.); zhushuyi@issp.ac.cn (S.Z.); hwzhang@issp.ac.cn (H.Z.); wpcai@issp.ac.cn (W.C.); 2 Science Island Branch of Graduate School, University of Science and Technology of China, Hefei 230026, China; 3 State Key Laboratory of NBC Protection for Civilian, Beijing 102205, China; 13651160976@139.com; 4 Dongying City Center for Disease Control and Prevention, Dongying 257000, China; dyjkljq@126.com; 5 Shandong Shouguang Testing Group Co., Ltd., Weifang 262700, China; wuwenguang2003@163.com

**Keywords:** Au/CuS hybrids, high-density nanotips, SERS, signal reproducible, substrate recyclable

## Abstract

Surface-enhanced Raman scattering (SERS) provides an unprecedented opportunity for fingerprinting identification and trace-level detection in chemistry, biomedicine, materials, and so on. Although great efforts have been devoted to fabricating sensitive plasmonic nanomaterials, it is still challenging to batch-produce a SERS substrate with high sensitivity, good reproducibility, and perfect recyclability. Here, we describe a facile fabrication of three-dimensional (3D) hierarchical Au/CuS nanocomposites, in which high-density Au nanotips enable highly SERS-active sensing, and the well-defined microflower (MF) geometry produces perfect signal reproducibility (RSD < 5%) for large laser spot excitations (>50 μm^2^), which is particularly suitable for practical on-site detection with a handheld Raman spectrometer. In addition, a self-cleaning ability of this Au/CuS Schottky junction photocatalyst under sunlight irradiation allows complete removal of the adsorbed analytes, realizing perfect regeneration of the SERS substrates over many cycles. The mass-production, ultra-sensitive, high-reproducibility, and fast-recyclability features of hierarchical Au/CuS MFs greatly facilitate cost-effective and field SERS detection of trace analytes in practice.

## 1. Introduction

Surface-enhanced Raman scattering (SERS) has emerged as a highly powerful spectroanalytical technique due to its competitive merits in fingerprinting identification, trace-level sensitivity, and nondestructive detection [1,2,3]. The Raman signal of the matter to be analyzed in SERS can be greatly amplified by several orders of magnitude and, in some cases, enables single-molecule detection [4,5,6,7,8]. The significant amplification mainly arises from the enormous enhancement of the electromagnetic (EM) field in the surface vicinity of coinage metal (typically Au, Ag, and Cu), which is generated by the localized surface plasmon resonances (LSPRs) upon specific laser excitation [9]. In general, it is believed that the EM enhancement is extremely strong at interparticle gaps and around sharp tips of metallic nanostructures, typically referred to as hotspots [10]. Consequently, the design, construction, and mass production of highly sensitive nanomaterials are key prerequisites for the trace detection of chemical and biological analytes, especially for commercial applications in the near future. To date, remarkable success in sensitivity has been achieved by fabricating diverse silver or gold geometrical nanostructures such as nanospheres [11], nanorods [12], nanostars [13,14,15], and nanourchins [16,17,18], which serve as basic building blocks for the sensing colloids or solid substrates. Among those architectures, the hierarchical nanostructures have attracted significant attention because of their ultimate enhancement factors (EFs) of 10^7^–10^9^, benefiting from the plentiful hotspots formed between nanoscopic gaps, corners, valleys, sharp protrusions, and crevices [19,20,21]. However, those anisotropic architectures were frequently prepared by wet-chemical procedures with protecting/capping agents such as malachite green isothiocyanate (MGITC), cetyltrimethylammonium bromide (CTAB), polyvinylpyrrolidone (PVP), and citrate ions to control the nucleation and crystal growth. Observable signal interferences from the residual agents commonly occur for such ultra-sensitive SERS substrates, accompanied with a significant decrease in sensitivity due to the hotspots’ occupying effect [22]. Alternatively, lithography-assisted patterning techniques combined with physical deposition were utilized to fabricate periodic hierarchical micro/nanoarrays with controllable dimension, morphology, and spacing in the sensing metals [23,24,25], which were totally clean for ultrasensitive surfaces, but it was difficult to achieve cost-effective mass production with them due to the sophisticated equipment and tedious procedure.

In addition to the sensitivity, the signal reproducibility of a SERS substrate is extensively crucial for reliable detection of trace analytes in practice, especially for quantitative analysis. In this regard, plenty of pioneered studies have also been conducted and have drawn enlightening suggestions, such as employing plasmonic materials with high chemical stability [26], fabricating nanostructures with well-defined geometry [27,28,29], and improving the uniformity of the entire substrates [30]. Accordingly, mass-produced Au hierarchical nanoarchitectures commonly show great advantages as basic sensitive units for reproducible detection in the SERS field. Even for the substrates obtained by random aggregation of the hierarchical units, improved signal reproducibility can be eventually achieved by using larger laser spots, which provides an averaging of massive analytes in the excitation volume [31].

Unlike the electrical and electrochemical gas sensors, people rarely mention the recoverability or recyclability of the sensing device in the field of SERS detection [32,33,34]. Researchers seem to have acquiesced that SERS substrates should be discarded after being used once, that is, they are not reusable. However, from the perspective of environmental protection and economy, enough attention should also be given to the regeneration of already used SERS devices. Recently, researchers have shown that composite SERS substrates consisting of plasmonic noble metals and photocatalytic oxides/sulfides can be regenerated by simple irradiation with UV/visible light, which enables effective removal of analytes adsorbed to the surfaces under photocatalytic degradation. For instance, a series of composite substrates such as ZnO/Ag nanoarrays [35,36], Au@CdS core–shell nanocomposites [37], and Au-coated TiO_2_ nanotube arrays [38] have been presented for repeated utilization in Raman assays even for tens of cycles. However, up to now, a SERS substrate simultaneously possessing a super signal amplification, high reproducibility, and perfect substrate recyclability is extremely challenging, which impedes the widespread use of SERS sensors in practical applications.

Herein, we present a facile solvent approach for the mass production of three-dimensional (3D) Au/CuS microflower (MF) hybrids with a highly roughened hierarchical structure. The individual Au/CuS MF is composed of cross-linked CuS nanopetals with curved surfaces, on which high-density Au nanotips (>1000/μm^2^) with 30–50 nm widths are loaded in a radial arrangement. The sharp protrusions, the narrow valleys of the hierarchical Au/CuS MFs, as well as the overlap between individual MFs for the self-assembled substrate provide abundant hotspots for SERS, hence showing a high enhancement capability at ~10^7^ magnitude and a detection limit of 10^−10^ M for Rhodamine 6G (R6G) probes, without any interference signals being observed. In addition, the hierarchical Au/CuS hybrid substrates demonstrate a perfect signal reproducibility (<5% standard deviation) upon large laser spot illuminations (>50 μm^2^), which is of special significance for the portable on-site detection with a handheld Raman spectrometer. More importantly, these hybrid substrates can be regenerated completely via degradation and removal of the surface-adsorbed analytes, and such a self-cleaning feature arises from photocatalysis of the underlying CuS basis of the nanocomposites. The facile and feasible strategy offers a robust avenue for the mass production of 3D hierarchical plasmonic/semiconducting nanocomposites, and the assembled substrate provides a competitive platform for sensitive, reproducible, and recyclable SERS detections.

## 2. Experimental

### 2.1. Materials and Reagents

Copper sulfate pentahydrate (CuSO_4_·5H_2_O), sodium hydroxide (NaOH), glucose, sodium sulfide (Na_2_S), chloroauric acid (HAuCl_4_), Rhodamine 6G (R6G), and crystal violet (CV) were purchased from Aladdin Chemical Reagent Co., Ltd. (Beijing, China). Deionized water with a resistivity of 18.2 MΩ·cm^−1^ (25 °C) was used for all solutions by a Millipore Milli-Q system. All the chemical reagents were directly used without further purification.

### 2.2. Synthesis of 3D Hierarchical Au/CuS MFs

**Figure 1:**
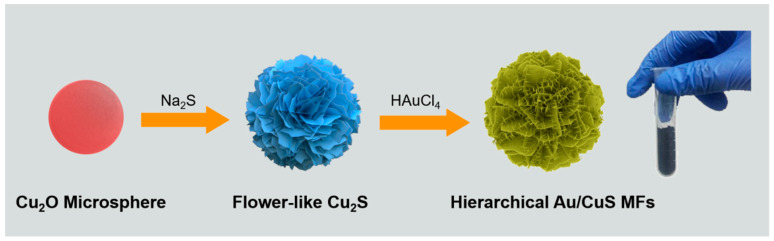
Schematic representation of the three-step solvent procedure for mass-production of 3D hierarchical Au/CuS MFs.

Nanotips-composed 3D hierarchical Au/CuS MFs were facilely mass-prepared through a three-step solvent approach under ambient conditions. The fabrication protocol is demonstrated in Figure 1. First, monodisperse Cu_2_O microspheres were prepared through a solvothermal reducing method, in which 50 mL of 1 M glucose solution was added into a freshly prepared Cu(OH)_2_ slurry (obtained by direct mixing of 3 M NaOH and CuSO_4_ solutions) and was maintained for 40 min at 50 °C. Then, nanosheet-composed flower-like Cu_2_S architectures were synthesized through an anion exchange method, simply by adding 1.0 g of Na_2_S powder into as-obtained Cu_2_O microsphere precursors at room temperature. Finally, nanotips-composed 3D hierarchical Au/CuS MFs were obtained by adding a 7.5 mL HAuCl_4_ (1 wt%) solution into the Cu_2_S colloidal solutions and were maintained for 30 min at 25 °C. All the intermediate/final products were collected by centrifugation, washed with deionized water three times to remove residual impurities, and re-dispersed into 100 mL of deionized water for further reaction/characterization.

### 2.3. Characterization

The morphologies of the intermediate/final products were observed by a field emission scanning electron microscope (FE-SEM, FEI Sirion 200, Hillsboro, OR, USA) equipped with an Energy-Dispersive X-ray Spectrometer (EDS). The crystal structure was analyzed using an X-ray diffractometer (XRD, the Philips X’Pert, Amsterdam, Holland) with copper Kα radiation (λ = 0.15406 nm) at room temperature. Transmission electron microscopy (TEM) and high-resolution transmission electron microscopy (HR-TEM) studies were performed on a JEM-200CX (Tokyo, Japan) operated at 200 kV. UV–vis absorption spectra were recorded on a spectrophotometer (Shimadzu UV-2600, Tokyo, Japan) in the wavelength range of 200–800 nm.

### 2.4. SERS Measurements

R6G and CV molecules were selected as a probe to evaluate the SERS activity of the 3D hierarchical Au/CuS MFs. Both molecules are positively charged and the clean noble metal surfaces are negatively charged. Accordingly, the CV and R6G molecules demonstrate perfect affinity to the surface through electrostatic interaction, which endows perfect SERS detection performances. The nanocomposites were soaked into R6G and CV solutions for 6 h, re-collected by centrifugation, and self-assembled on the silicon wafer (3 mm × 3 mm) by the spin-coating method for further SERS measurements. In addition, films consisting of pure Au nanoparticles (NPs) 50 nm in diameter were also prepared as the control group. SERS spectra were acquired by a confocal microprobe Raman spectrometer (Renishaw inVia, 532 nm laser line) with a laser power of 0.5 mW and acquisition time of 5 s for all measurements.

To evaluate the recyclability of the Au/CuS hybrid SERS substrates, the used sensing substrate was irradiated under simulated sunlight (a xenon lamp with a 50 W illumination power) to degrade the as-adsorbed R6G molecules followed by repeated adsorption and Raman examination.

## 3. Results and Discussion

### 3.1. Morphology and Structure

**Figure 2:**
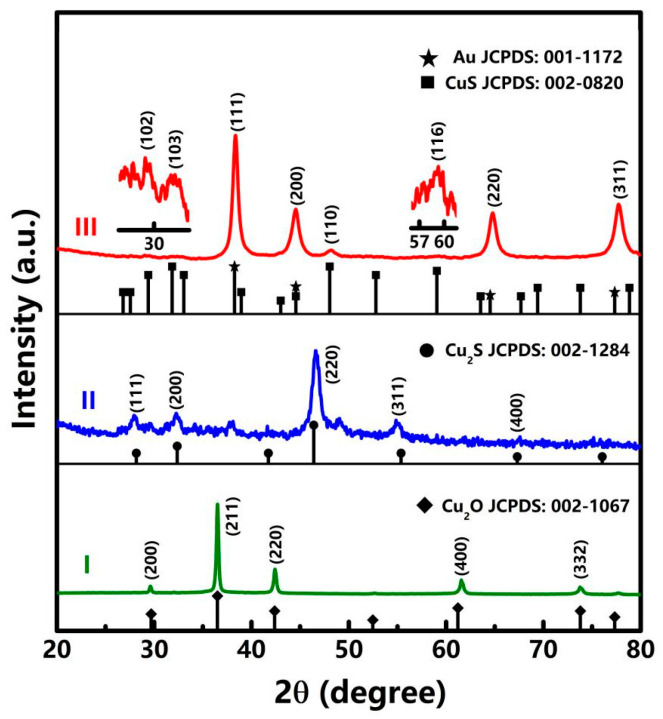
XRD patterns of the two intermediates (curve I and II) and final product (curve III) facilely mass-produced by the solvent approach.

For one typical fabrication, gram-level final products can be readily obtained. During these solvent procedures, the color of the obtained colloids was changed from brick red and black to brown. Figure 2 demonstrates XRD patterns of the two intermediates and the final product, in which the enormous differences in diffraction peaks reveal distinct crystalline phases for each product. Typically, for the two intermediates (curve I and II), the diffraction peaks can be ascribed to the cubic Cu_2_O (JCPDS No. 002-1067) and Cu_2_S (JCPDS No. 00-002-1284) phases, indicating the ionic replacement process. After addition of HAuCl_4_, the predominant strong peaks at 38.2°, 44.7°, 64.6°, and 77.9° are well matched with (111), (200), (220), and (311) planes of the face-centered-cubic (FCC) gold (curve III, JCPDS No. 001-1172), respectively. Meanwhile, weaker diffraction peaks at 29.2°, 33.1°, 48.3°, and 59.1° can be well assigned to (102), (103), (110), and (116) planes of CuS (JCPDS No. 002-0820), respectively. Accordingly, a redox reaction between HAuCl_4_ and Cu_2_S occurred, which led to a binary mixture of crystalline Au/CuS substances.

**Figure 3:**
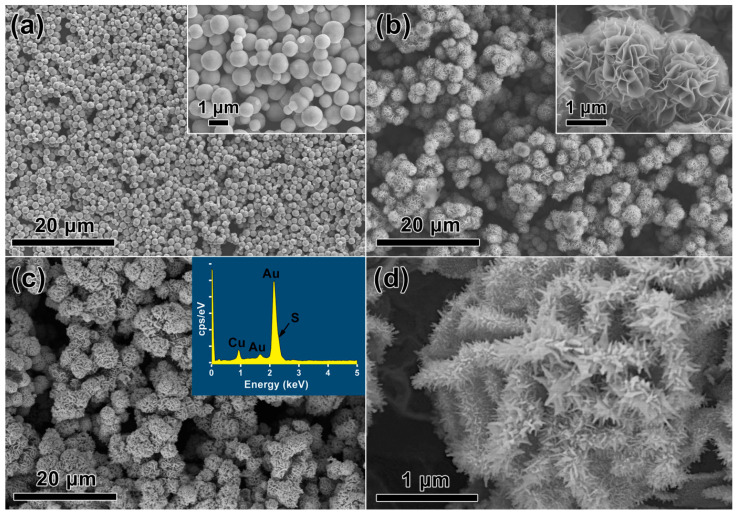
FE-SEM images of the (**a**) Cu_2_O, (**b**) Cu_2_S intermediates, and the (**c**,**d**) final Au/CuS nanocomposite. Insets in (**a**,**b**) correspond to magnified observations, and (**c**) EDS analysis.

FE-SEM observations were also conducted to characterize the morphologies of individual products (Figure 3). It showed that the initial Cu_2_O intermediates are well-defined monodisperse microspheres with a mean dimension of, ca., 1 μm (Figure 3a). After ionic replacement, the microspheres transform to hierarchical flower-like Cu_2_S microstructures, which are composed of numerous curving nanopetals with a uniform thickness of, ca., 35 nm (Figure 3b). After redox reaction by adding HAuCl_4_, the microflower structure is almost retained but with a larger particle dimension of, ca., 3 μm for the final product (Figure 3c). The corresponding EDS analysis (inset in Figure 3c) reveals the elemental compositions of Au, Cu, and S, which is consistent with the XRD results and confirms the binary chemical composition for the Au/CuS MFs. The magnified FE-SEM observation of an individual microflower clearly reveals that there exist a considerable number of Au nanotips on the surface of nanopetals (Figure 3d). The Au nanotips are about 300 nm in length, 30–50 nm in width, >1000/μm^2^ in density, and present a radial arrangement on curved nanopetals, resulting in a high-density-nanotips-composed 3D hierarchical MF structure.

**Figure 4:**
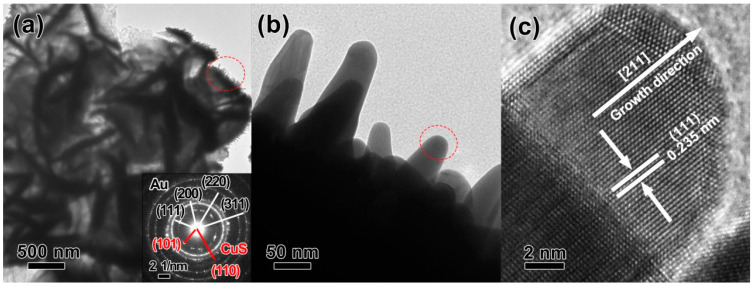
TEM and HR-TEM observations of 3D hierarchical Au/CuS MFs. (**a**) Low-magnified image, (**b**) enlarged observation, and (**c**) the HR-TEM images of an isolated nanotip. The inset in (**a**) is the corresponding SAED pattern of the circled region, and those ascribed to CuS phase are marked in red.

Figure 4 shows representative TEM and HR-TEM images of the 3D hierarchical Au/CuS MFs product. The image contrast in low magnification (Figure 4a) confirms its petal-consisting hierarchical structure, and nanosized tips can be clearly observed on the curved petals. The corresponding SAED pattern acquired from the local area of the petal also demonstrates diffraction rings ascribed to crystalline Au and CuS phases (inset of Figure 4a). An enlarged image (Figure 4b) further confirms that the well-defined nanotips with an arc-shaped top are distributed on thick petal platforms along different directions. HR-TEM observation was further carried out to evaluate the crystallinity and orientation features of an isolated nanotip, as shown in Figure 4c. The clear 2D ordered lattice fringes reveal its perfect crystallinity, and the d-spacings of 0.238 nm correspond to the (111) facet of cubic gold. We can also conclude that the growth direction of the nanotip is along the direction of <211> by analyzing the lattice fringes exposed on the nanotip surface.

### 3.2. Influence Factors

The 3D Au/CuS hierarchical MFs are formed in the hydrothermal redox reaction between HAuCl_4_ and Cu_2_S. Accordingly, the experimental conditions including the amount of added HAuCl_4_ as well as the reaction temperature are crucial to optimizing the hierarchical nanostructure and exploring its formation mechanism.

#### 3.2.1. Amount of HAuCl_4_ Solution

**Figure 5:**
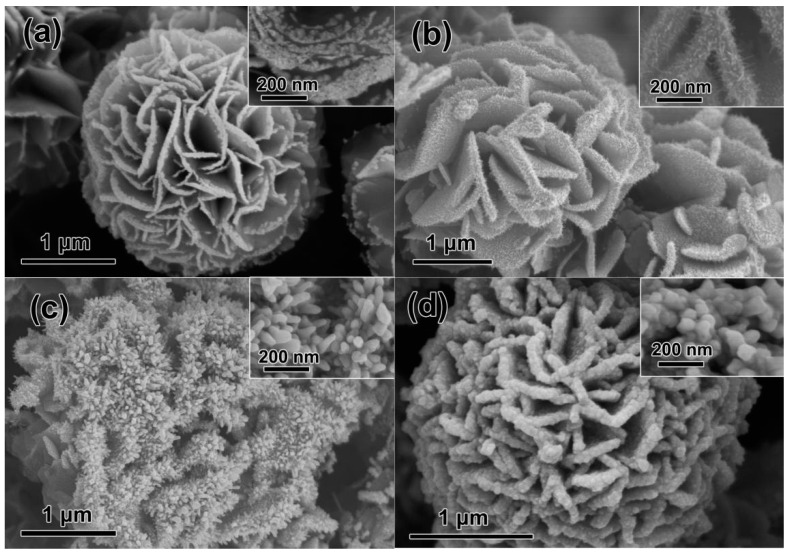
Morphological evaluations of the 3D hierarchical Au/CuS nanostructures obtained by adding different amounts of HAuCl_4_ into 15 mL of Cu_2_S microflower suspension. (**a**) 0.2 mL, (**b**) 1 mL, (**c**) 10 mL, and (**d**) 15 mL. Insets are the corresponding magnified images.

For a given Cu_2_S colloidal precursor (typically 15 mL), HAuCl_4_ solutions with a stepped incremental content (0.2~15.0 mL) were individually added to investigate the evaluation of the final products. As demonstrated in Figure 5, it is obvious that the amounts of decorated Au on the curved petal surfaces are significantly increased with the increase in HAuCl_4_, which is confirmed by the XRD investigation (Figure S1). However, there exist significant differences in the distribution and morphology of the Au decorations for these cases. In detail, for a low adding amount (typically 0.2 mL), ultrafine Au NPs instead of nanotips are preferentially decorated to the edges of curved petals (Figure 5a), and a decremental NPs coverage from the outside edge to the interior surfaces is readily observed. In addition, with the addition of slightly increased HAuCl_4_ (1.0 mL), uniform Au nanoneedles, ca., 10 nm in width and 60 nm in length are covered on the entire surface of curved petals (Figure 5b). Meanwhile, for excess HAuCl_4_ addition cases (typically 10 and 15 mL), Au nanorods (80 nm in diameter, Figure 5c) and even compact Au films (Figure 5d) are obtained.

#### 3.2.2. Reaction Temperature

**Figure 6:**
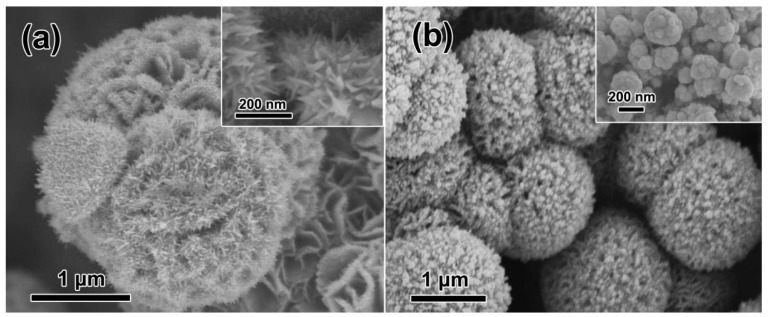
FE-SEM observations of the Au/CuS products obtained by adding 7.5 mL of HAuCl_4_ solution into 15 mL of Cu_2_S microflower suspension at different temperatures. (**a**) 5 °C and (**b**) 50 °C. The insets are the corresponding magnified images.

The reaction temperature is known as a key factor in the hydrothermal fabrication of nanostructures by influencing the nucleation and subsequent crystal growth processes. Accordingly, additional experiments at both a lower (5 °C) and higher (50 °C) temperature were individually conducted for the fabrication of the hierarchical Au/CuS architectures, as illustrated in Figure 6. Typically, sharper Au nanoflakes are obtained at a lower reaction temperature (Figure 6a). However, compact Au films consisting of NPs, ca., 200 nm in diameter are covered on the petals at a higher reaction temperature (Figure 6b). It is concluded that the temperature remarkably influences the formation of an Au nucleus in the redox reactions as well as the subsequent crystal growth of Au nanostructures. In addition, only a moderate temperature environment can lead to high-density-nanotips-composed Au/CuS hierarchical MFs.

### 3.3. Formation Mechanism

**Figure 7:**
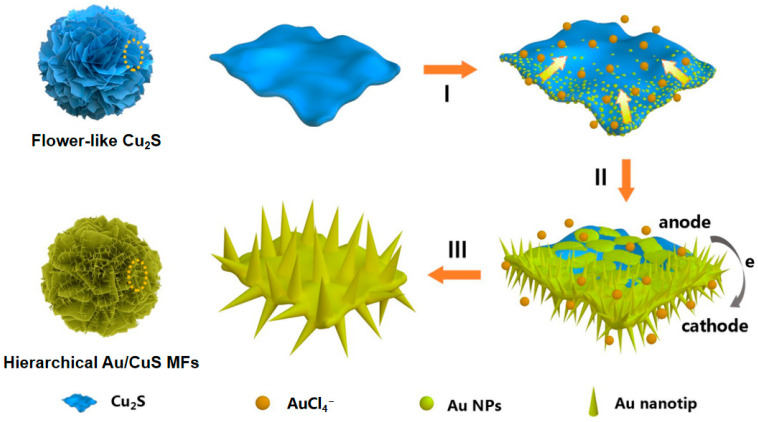
Schematic illustration of the formation mechanism for high-density-nanotips-composed 3D hierarchical Au/CuS MFs.

Based on these results, we propose that the high-density-nanotips-composed 3D hierarchical Au/CuS MFs are formed based on a synergistic growth mechanism (Figure 7), namely a redox-dominated surface nucleation and subsequent galvanic deposition process, which leads to the overspreading of Au NPs on the CuS base and radial growth of Au nanotips perpendicular to the surfaces, respectively. For the redox reaction, the standard electrode potential of a AuCl_4_^−^/Au pair (1.002 V vs. the standard hydrogen electrode (SHE), 25 °C) is much higher than that of a CuS/Cu_2_S pair (0.53 V vs. SHE, 25 °C) [39,40], which would induce the reduction of the aqueous AuCl_4_^−^ to Au nucleus on the Cu_2_S surface (I in Figure 7). This process can be represented by the following equation:

2AuCl_4_^−^ + 3Cu_2_S = 2Au + 3CuS + 3Cu^2+^ + 8Cl^−^ (1)

Such a redox reaction would preferentially occur on the Cu_2_S petal edges, which can provide more abundant defects and unsaturated bonds. Then, the redox-dominated gold nucleation gradually spreads to the central region of the petal. Such a successive process can be verified through TEM observations of the products obtained at different reaction durations (Figure S2).

Once the Au NPs are dispersedly loaded on the Cu_2_S petals by the redox reaction, isometrical galvanic cell microsystems are readily formed, each of which consists of a Cu_2_S nanosheet as the anode and Au NP as the cathode. Such a process can be described by the following equations:

Cu_2_S = CuS + Cu^2+^ +2e   (anode) (2)

AuCl_4_^−^ + 3e = Au + 4Cl^−^    (cathode) (3)

It indicates that the interior Cu_2_S below the Au NPs can be further transformed to CuS through the anodic reaction in the galvanic cell. Meanwhile, the cathodic reaction promotes continuous growth of the Au NPs along the longitudinal direction, resulting in single crystalline Au nanotips with a preferred <211> orientation.

For both the redox and cathodic reactions, the supply and migration rate of reactive AuCl_4_^−^ significantly affect the radial growth of Au nanotips, which has been confirmed by the fact that the morphology and aspect ratio of the Au nanotips can be effectively modulated by adjusting the adding amount of HAuCl_4_ or reaction temperature.

Based on the above synergistic growth mechanism, the surface of CuS micropetals will be gradually covered with films composed of Au NPs and finally transformed to high-density-nanotips-composed hierarchical Au/CuS microflowers.

### 3.4. SERS Performance

#### 3.4.1. High SERS Sensitivity

**Figure 8:**
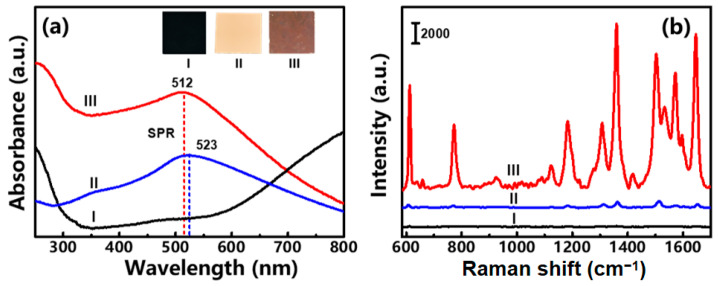
(**a**) Optical images and absorption spectra of the sensing substrates consisting of (I) CuS MFs, (II) Au NPs, and (III) Au/CuS hybrid MFs. (**b**) The acquired SERS spectra of substrates with exposure to 10^−7^ M R6G molecules.

For SERS performance evaluation, the as-prepared 3D hierarchical Au/CuS MFs were re-dispersed into ethanol, and subsequently spin-coated onto a silicon substrate to construct self-assembled sensing films with a thickness of several micrometers. In addition, pure CuS and Au NP films (Figure S3) with the same thicknesses were prepared as counterparts. The optical images and absorption spectra of those sensing substrates were compared in Figure 8a. It indicates that the Au/CuS hybrids substrate typically shows a brown color, and in addition to the strong valence-to-conduction band transition absorption in the UV region, a broad absorption peak around 512 nm is also observed, arising from the LSPR of Au nanotips. However, the optical absorption of the 3D hierarchical Au/CuS MFs decreases in the near-infrared region. This is because the optical absorption in the near-infrared region mainly comes from the LSPR effect of the vacancy in the Cu_(2−x)_S [41]. As the frequency of this LSPR depends on the number of carriers in the semiconductor species [42], different adding amounts of chloroauric acid will affect the x value and the number of carriers in Cu_(2−x)_S species (Figure S4), resulting in a decrease in the near-infrared region. Accordingly, a 532 nm laser should be chosen as an optimal excitation for a better Raman enhancement performance, which is confirmed by a series of excitation-dependent SERS detection experiments (Figure S5). The SERS assays reveal that, for 10^−7^ M R6G probes, the Au/CuS hybrid substrate demonstrates significantly enhanced Raman signals compared with the semiconducting CuS MFs and pure plasmonic Au NPs without any interference vibrations (Figure 8b). In addition, it was found that the 3D hierarchical Au/CuS MFs obtained by adding 7.5 mL of HAuCl_4_ solution have a better SERS activity than the counterparts do (Figure S6). Thus, such Au/CuS MFs were chosen as a typical substrate to evaluate the SERS performances. Meanwhile, the calculated enhancement factor (EF, see details in the Supporting Information and Figure S7) for the Au/CuS hybrid substrate is 4.4 × 10^7^, which is two orders of magnitude higher than that of the Au NPs counterpart (2.8 × 10^5^).

**Figure 9:**
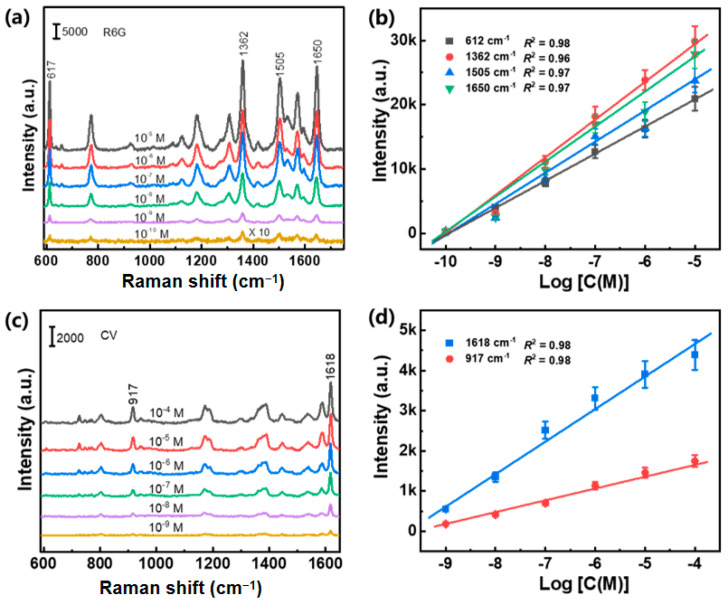
The concentration-dependent SERS spectra of R6G (**a**) and CV (**c**) acquired from the Au/CuS hybrid substrate. (**b**,**d**) The linear fitting of the SERS signal intensities of R6G at 612, 1362, 1505, and 1650 cm^−1^ and CV at 917 and 1618 cm^−1^ versus the logarithmic R6G and CV concentration. *R* is the correlation coefficient. Error bars were calculated from 20 different spectra.

Furthermore, we explored the detection limit of the Au/CuS hybrid substrate and the correlations between the peak intensities and analyte concentrations (Figure 9). It is further verified that the sensitivity of the substrate is extremely high, where R6G molecules (10^−10^ M) and CV molecules (10^−9^ M) can be readily tracked. As displayed in Figure 9b,d, the prominent peaks of R6G at 612, 1362, 1505, and 1650 cm^−1^ (CV at 917 and 1618 cm^−1^) were chosen as the identification position for the quantitative analysis of R6G (CV), and the intensities of almost all the characteristic peaks concurrently increase as the analyte concentration gradually increases to 10^−5^ M (R6G) or 10^−4^ M (CV). All the calibration curves between intensities of vibrational peaks and logarithmic concentrations reveal perfect linear relationships with *R*^2^ > 0.96. Accordingly, the high sensitivity together with the perfect linear dependence of the hybrid sensing substrate will provide a very convenient and straightforward analytical strategy for quantitative detection of trace analytes.

#### 3.4.2. Improved Repeatability

**Figure 10:**
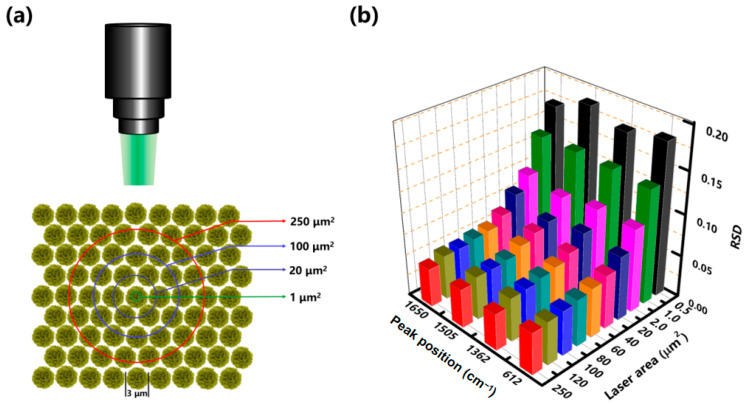
The SERS signal reproducibility evaluation of 3D hierarchical Au/CuS hybrid substrate. (**a**) Schematic illustration of SERS measurements with different LSAs on the hybrid substrate by confocal microprobe Raman spectrometer. (**b**) The RSD values of the four characteristic peaks of R6G molecules at 621, 1362, 1505, and 1650 cm^−1^ obtained with different LSAs.

In terms of practical SERS detection, reliable qualitative and quantitative analysis requires a high reproducibility of Raman signals acquired from area-to-area and substrate-to-substrate. From this perspective, the Au/CuS hybrid substrate consisting of micron-sized hierarchical MFs should possess a poor repeatability performance owing to the structural anisotropy of the sensing unit [43]. However, based on the fact that the read-out signals come from the overall contributions of plasmonic nanostructures covered by the laser spot areas (LSAs), an improved reproducibility will be obtained for large LSAs case due to the averaging of massive analytes in the excitation volume. In the present work, the LSAs were adjusted from 0.5 to 250 μm^2^ by using different-numerical-aperture lenses and controlling the laser focus on a confocal microprobe Raman spectrometer (Figure 10a). It is demonstrated that poor signal reproducibilities (RSD up to 18%) are obtained for the LSAs with comparable dimensions (typically 0.5, 1.0, and 2.0 μm^2^) to that of the hierarchical Au/CuS MF (Figure 10b). However, the deviation rapidly decreases with the increase in the area of spots from 2.0 to 40 μm^2^, and low values (RSD < 5%) are obtained for LSAs larger than 60 μm^2^. As the widely used handheld Raman spectrometers commonly possess spot areas on the order of hundreds of square microns [44,45], the high repeatability feature will facilitate construction of on-site sensing devices for both cost-effective and reliable SERS detection of trace analytes.

#### 3.4.3. Environmental Adaptability

**Figure 11:**
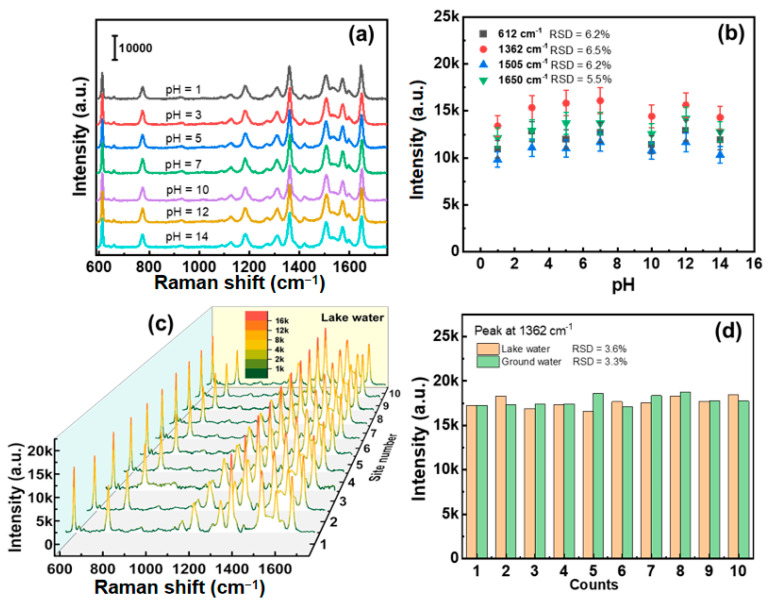
(**a**) SERS spectra acquired from the Au/CuS hybrid substrate immersed in 10^−7^ M R6G solutions with pH values ranging from 1 to 14. (**b**) The absolute peak intensities at 612, 1362, 1505, and 1650 cm^−1^ versus the pH values (data from (**a**)). (**c**) SERS spectra acquired from the Au/CuS hybrid substrate immersed in lake water (the Dongpu Lake in Hefei, China) with spiked 10^−7^ M R6G on a handheld Raman spectrometer. (**d**) The histograms for the peak intensities of R6G at 1362 cm^−1^ (data from (**c**) and Figure S8a).

**Figure 12:**
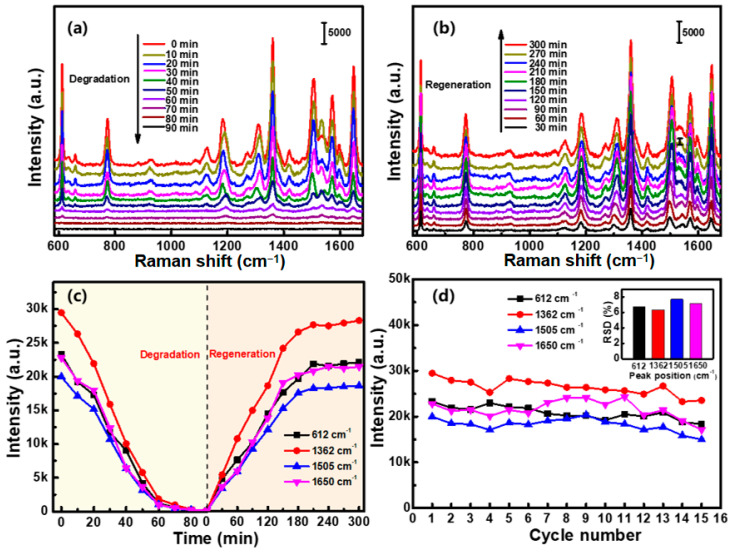
The recyclability of the 3D hierarchical Au/CuS MFs substrate: (**a**) SERS spectra acquired from the substrate irradiated by the simulated sunlight with different time durations. The substrate was initially soaked in 10^−7^ M R6G solution for 360 min. (**b**) Spectra of recyclable substrate by re-immersing in R6G solution with the same concentration. (**c**) The intensity variations of the four characteristic peaks of R6G molecule in degradation and regeneration process. (**d**) The intensity variations of R6G molecule acquired from the substrate at different reusage cycles. The inset is the finally obtained RSD values.

The SERS activity of the 3D hierarchical Au/CuS hybrid substrate under different pH conditions can remain stable, which is crucial for the field detection. The SERS spectra obtained by immersing the Au/CuS hybrid substrate in 10^−7^ M R6G solutions with different pH values are shown in Figure 11a. Although the pH value has a wide range of changes from 1 to 14, the obtained SERS signal intensities remain stable and the RSD values of the characteristic peak intensity (612, 1362, 1505, and 1650 cm^−1^) are less than 6.5% (Figure 11b). Due to the high repeatability feature and perfect environmental adaptability of the Au/CuS hybrid substrate, a simulated on-site detection of R6G for natural water was conducted on a handheld Raman spectrometer with a laser spot of 0.2 mm^2^. We added R6G molecules to the lake (the Dongpu Lake in Hefei, China) and ground water before testing, in which the spiked concentrations of both were 10^−7^ M. The SERS spectra (Figure 11c or Figure S8a) taken from 10 randomly selected points in the R6G spiked actual water were comparable with that obtained in the R6G spiked laboratory water (Figure 8b), and the calculated RSD values of peak intensities at 1362 and 612 cm^−1^ were less than 5% (Figure 12c or Figure S8b), which confirms the above results that the Au/CuS hybrid substrate is an ideal candidate for on-site reliable detection of trace analytes in combination with a handheld Raman spectrometer.

#### 3.4.4. Perfect Recyclability

The recyclability is another crucial yet rarely reported aspect in chemical detection using SERS devices, which leads to a more cost-effective and eco-friendlier assay. It has been previously reported that thermal desorption and photocatalytic degradation are feasible routes to regenerate the used substrates by exfoliating the adsorbed analytes from metal surfaces [34]. However, as the heat-induced melting or diffusion of surface atoms commonly leads to reshaping the plasmonic substrates, which significantly decreases the Raman activity, photodegradation has been regarded as the optimal way for substrate regeneration. In present work, the sensing substrates are composed of hierarchical Au/CuS nanocomposites, in which the semiconducting CuS with a band gap of ~0.75 eV has a high photocatalytic activity by generating electron–hole pairs even under visible-light irradiation [46], and the metal–semiconductor Schottky junction further increases the life-time of electron–hole pairs [47], which is favorable for improving the photocatalytic performances. As demonstrated in Figure 12a, strong SERS signals were initially obtained for the Au/CuS hybrid substrate after exposure to a 10^−7^ M R6G solution. In addition, all the characteristic peaks gradually decreased with continuous irradiation by simulated sunlight, indicating that the R6G molecules were undergoing photocatalytic degradation on the surface. For an irradiation period of 80 min, the degradation rate almost reached 100%, and no vibrational peaks could be observed. When this substrate was recycled for SERS detection of R6G molecules with same concentration, it revealed that all the characteristic peaks were observed again and the intensities gradually increased with the prolonging of immersion time. When the soaking time reached 210 min (less than the previous time duration), the intensity of the characteristic peaks was almost equal to that of the totally new substrate (Figure 12b,c). In addition, such a perfect recyclable performance could be obtained even after repeated use for 15 times, in which the RSD of Raman signals was less than 8.0% (Figure 12d). Moreover, there was no obvious change in morphology of the Au/CuS hybrid substrate before and after 15 rounds of testing (Figure 3d and Figure S9).

## 4. Conclusions

In summary, we have developed a facile solvent approach for the mass production of high-density-nanotips-composed hierarchical Au/CuS MFs nanocomposites. A highly SERS-active sensing substrate was fabricated by assembling the Au/CuS hybrid nanoarchitectures, where the sensitivity was two orders of magnitude higher than that of traditional plasmonic Au NPs. Upon large laser spot excitations (LSAs > 50 μm^2^), the hybrid sensing substrate also demonstrated perfect signal reproducibility (RSD < 5%), which makes it an ideal candidate for the on-site reliable detection of trace analytes on a handheld Raman spectroscopy platform. Furthermore, the photocatalytic activity arising from the semiconducting CuS and Schottky junction in Au/CuS hybrids could be used for complete removal of the surface-adsorbed analytes, resulting in perfect regeneration and reuse of the SERS substrates over many cycles without a significant decrease in sensitivity. The mass-production, ultra-sensitivity, high-repeatability, and self-cleaning features of hierarchical Au/CuS MFs facilitate cost-effective and field SERS detection of trace analytes in practice.

## Supplementary Materials

The following supporting information can be downloaded at: https://www.mdpi.com/article/10.3390/nano12142359/s1. Figure S1: The normalized XRD spectrum of the products obtained after adding (I) 0.2, (II) 1, (III) 10, and (IV) 15 mL of HAuCl_4_ solutions (1 wt%) into 15 mL of flower-like Cu2S microparticles suspension; Figure S2: TEM images of the petals of Au/CuS nanocomposites obtained at reaction durations of (a) 5 min and (b) 10 min, respectively; Figure S3: A typical FESEM images of Au/CuS MFs, flower-like Cu_2_S microstructures and Au nanoparticles substrate, respectively; Figure S4: Optical absorption spectra of the 3D hierarchical Au/CuS nanostructures obtained by adding different amounts of HAuCl_4_; Figure S5: The Raman spectra of 10^−7^ M R6G solutions acquired from the Au/CuS hybrid substrate at excitation wavelengths of 532, 633 and 785 nm, respectively; Figure S6: (a) SERS spectra acquired from the Au/CuS hybrid substrate obtained by adding different amount of HAuCl_4_. (b) The absolute peak intensities at 612, 1362, 1505 and 1650 cm^−1^ versus the adding different amount of HAuCl_4_; Figure S7: The R6G Raman spectra of 50 µL 10^−9^ M on the hierarchical Au/CuS hybrid substrates and 50 µL 5 × 10^−3^ M on silicon wafer. The spectra are used to calculate the enhancement factor of Au/CuS hybrid substrates; Figure S8: (a) SERS spectra acquired from the Au/CuS hybrid substrate immersed in ground water with spiked 10^−7^ M R6G on a handheld Raman spectrometer. (b) The histograms for the peak intensities of R6G at 612 cm^−1^ [data from (a) and Figure 11c]; Figure S9: A typical FESEM image of the hierarchical Au/CuS hybrid substrates after 15 rounds of testing. Supporting Information: The XRD pattern of products obtained by adding different HAuCl_4_ solutions, TEM observations of the semi-products of Au/CuS obtained at different reaction durations, FESEM image of Au NP film, optical absorption spectra of the 3D hierarchical Au/CuS nanostructures obtained by adding different amounts of HAuCl_4_, SERS spectra acquired at different excitation wavelengths, SERS spectra acquired from the Au/CuS hybrid substrate obtained by adding different amounts of HAuCl_4_, calculation process for enhancement factor of the SERS substrates, SERS spectra acquired from the Au/CuS hybrid substrate immersed in R6G spiked ground water, and FESEM image of the hierarchical Au/CuS hybrid substrates after 15 rounds of testing. This material is free of charge on the internet.
