# Supplementary material for: High-Density-Nanotips-Composed 3D Hierarchical Au/CuS Hybrids for Sensitive, Signal-Reproducible, and Substrate-Recyclable SERS Detection
Source: Nanomaterials (Basel). 2022 Jul 10;12(14):2359. doi: 10.3390/nano12142359 (PMC9318914; doi:10.3390/nano12142359)
Supplement: Supplementary file 1 [file nanomaterials-12-02359-s001.zip › nanomaterials-1770373-supplementary.pdf]

Supporting Information

High-Density-Nanotips-Composed 3D Hierarchical Au/CuS Hybrids for Sensitive, Signal-Reproducible, and Substrate-Recyclable SERS Detection

Hao Fu[a, b,†], Weiwei Liu[c,†], Junqing Li[d], Wenguang Wu[e], Qian Zhao[a, *] Haoming Bao[a], Le Zhou[a], Shuyi Zhu[a, b], Jinglin Kong[c, *], Hongwen Zhang[a], and Weiping Cai[a, b]

[a]*Key Laboratory of Materials Physics, Institute of Solid State Physics, HFIPS, Chinese Academy of Sciences, Hefei 230031, China*

[b]*University of Science and Technology of China, Hefei 230026, China*

[c]*State Key Laboratory of NBC Protection for Civilian, Beijing 102205, China*

[d]*Dongying City Center for Disease Control and Prevention, Dongying 257000, China*

[e]*Shandong Shouguang Testing Group Co., Ltd., Weifang 262700, China*

† Those contributed equally to this work.

* To whom all correspondence should be addressed

E-mail: zhaoqian@issp.ac.cn; jlkong@sina.com

Figure S1 Fu Hao et.al

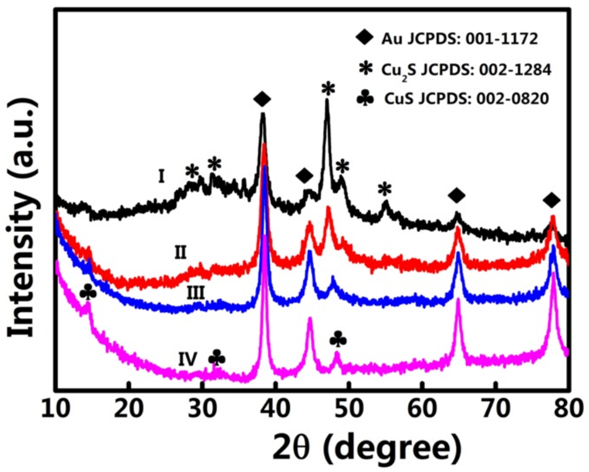

Figure S1. The normalized XRD spectrum of the products obtained after adding (I) 0.2, (II) 1, (III) 10, and (IV) 15 mL of $HAuCl_4$ solutions (1 wt%) into 15 mL of flower-like Cu_2S microparticles suspension.

Figure S2 Fu Hao et.al

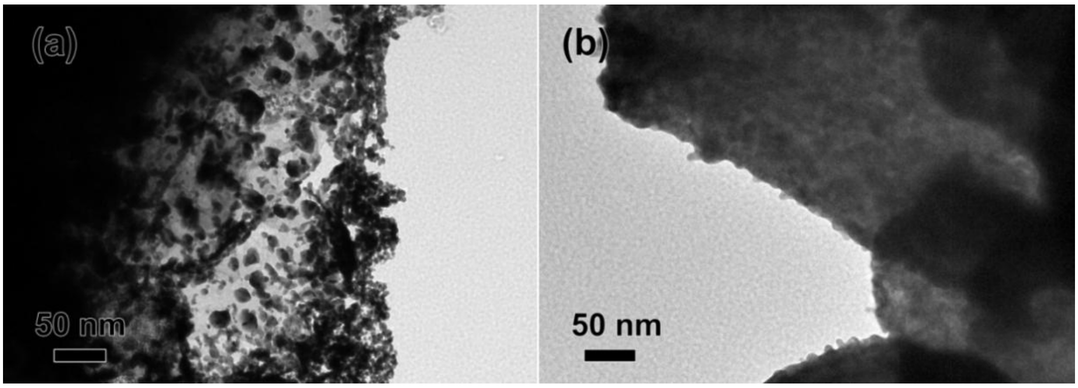

Figure S2. TEM images of the petals of Au/CuS nanocomposites obtained at reaction durations of (a) 5 min and (b) 10 min, respectively.

Figure S3 Fu Hao et.al

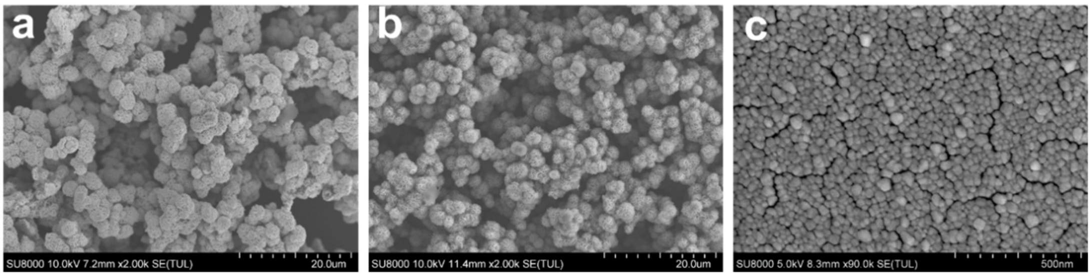

Figure S3. A typical FESEM images of Au/CuS MFs, flower-like Cu_2S microstructures and Au nanoparticles substrate, respectively.

Figure S4 Fu Hao et.al

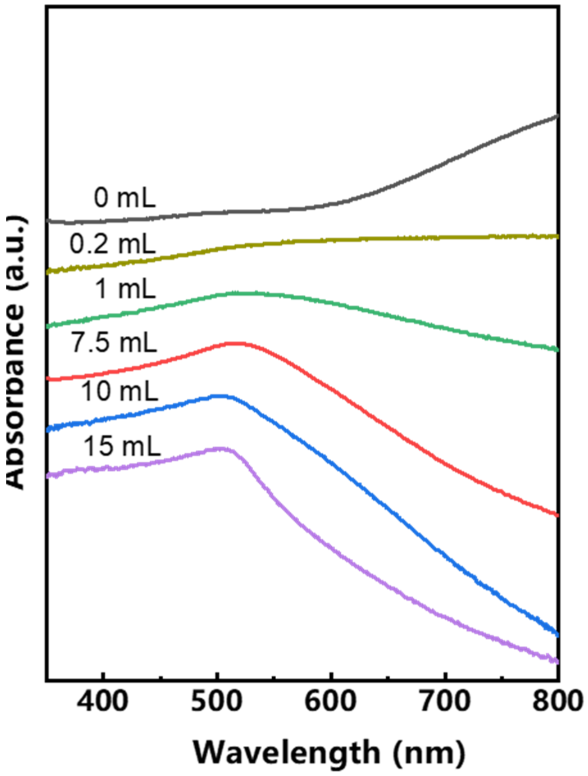

Figure S4. Optical absorption spectra of the 3D hierarchical Au/CuS nanostructures obtained by adding different amounts of $HAuCl_4$.

Figure S5 Fu Hao et.al

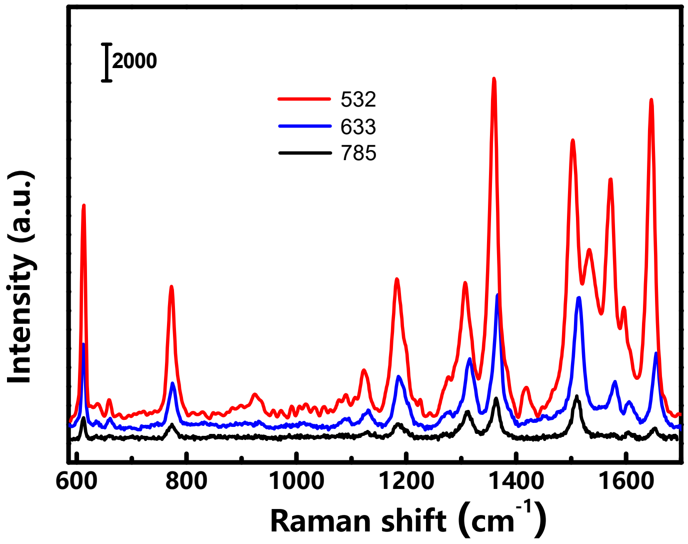

Figure S5. The Raman spectra of 10^{-7} M R6G solutions acquired from the Au/CuS hybrid substrate at excitation wavelengths of 532, 633 and 785 nm, respectively.

Figure S6 Fu Hao et.al

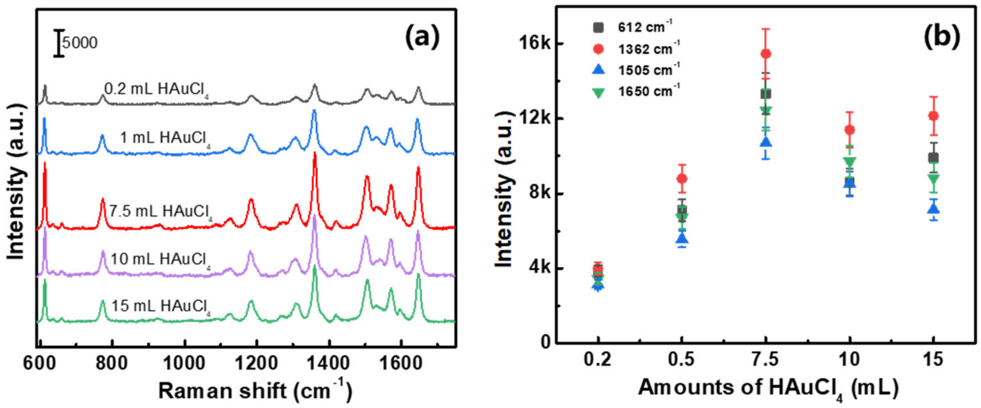

Figure S6. (a) SERS spectra acquired from the Au/CuS hybrid substrate obtained by adding different amount of $HAuCl_4$. (b) The absolute peak intensities at 612, 1362, 1505 and 1650 cm^{-1} versus the adding different amount of $HAuCl_4$.

Figure S7 Fu Hao et.al

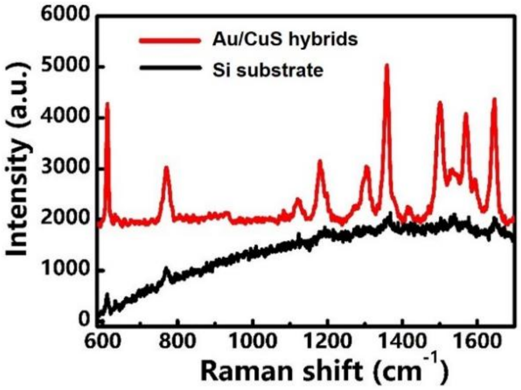

Figure S7. The R6G Raman spectra of 50 μL 10^{-9} M on the hierarchical Au/CuS hybrid substrates and 50 μL 5 x 10^{-3} M on silicon wafer. The spectra are used to calculate the enhancement factor of Au/CuS hybrid substrates.

Calculation of Enhancement Factor in SERS

The EF of the hierarchical Au nanostructures substrate was determined by computing the ratio of SERS to normal Raman scattering (NRS) of R6G using the following expression

$$EF = \frac{I_{SERS}/N_{SERS}}{I_{NRS} / N_{NRS}}$$

where I_{SERS} and I_{NRS} correspond to the integrated SERS and NRS intensities of R6G main peak at 612 cm^{-1}, respectively. N_{SERS} and N_{NRS} are the molecular numbers of different concentrations (50 μL 10^{-9} M and 50 μL $5x10^{-3}$ M) R6G in the SERS and NRS measurements. The calculated EF values Au nanostructure as high as $4.4x10^{7}$.

Figure S8 Fu Hao et.al

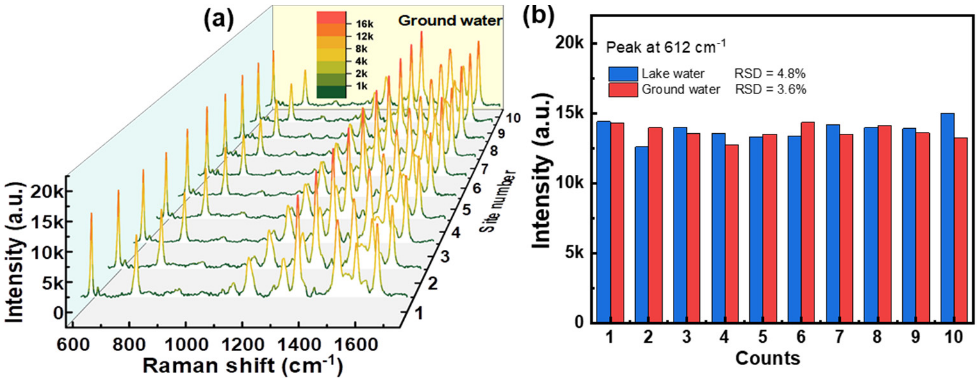

Figure S8. (a) SERS spectra acquired from the Au/CuS hybrid substrate immersed in ground water with spiked 10^{-7} M R6G on a handheld Raman spectrometer. (b) The histograms for the peak intensities of R6G at 612 cm^{-1} [data from (a) and Figure 11c].

Figure S9 Fu Hao et.al

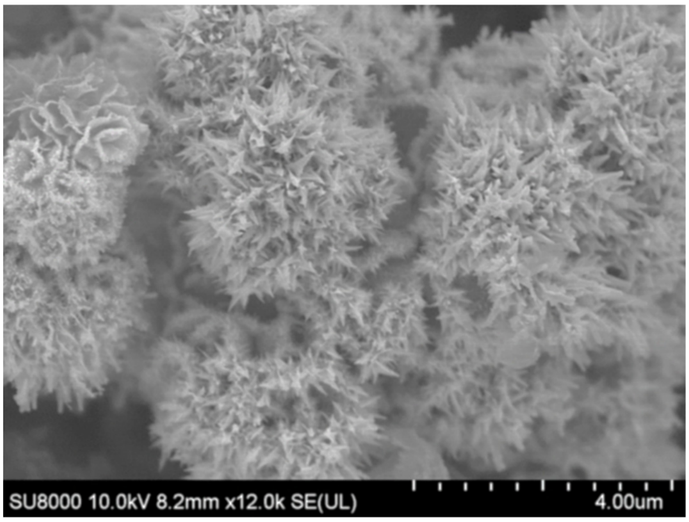

Figure S9. A typical FESEM image of the hierarchical Au/CuS hybrid substrates after 15 rounds of testing.
